# Maternal and Environmental Drivers of Trace Mineral Dynamics in Camel Dams and Neonates Across Regions and Seasons in Saudi Arabia

**DOI:** 10.3390/life15111730

**Published:** 2025-11-10

**Authors:** Mutassim M. Abdelrahman, Ibrahim A. Alhidary, Ahmad A. Aboragah, Mohammed M. Qaid, Mohammed A. Al-Badwi, Abdulkareem M. Matar, Mohsen M. Alobre, Ramzi A. Amran, Riyadh S. Aljumaah

**Affiliations:** 1 Department of Animal Production, College of Food and Agriculture Sciences, King Saud University, Riyadh 11451, Saudi Arabia; ialhidary@ksu.edu.sa (I.A.A.); aaboragah@ksu.edu.sa (A.A.A.); malbadwi@ksu.edu.sa (M.A.A.-B.); abdmatar@ksu.edu.sa (A.M.M.); malobre@ksu.edu.sa (M.M.A.); rjumaah@ksu.edu.sa (R.S.A.); 2 Department of Zoology, College of Science, King Saud University, P.O. Box 2455, Riyadh 11451, Saudi Arabia; ramran@ksu.edu.sa

**Keywords:** dromedary she-camel, environmental reservoirs, maternal–neonatal transfer, Saudi Arabia, seasonal-regional variations, temperature–humidity index, trace elements

## Abstract

Background: Dromedary camel in Saudi Arabia thrive across diverse desert ecosystems where trace minerals are vital for key physiological functions, yet data on how regional and seasonal factors affect these minerals in dams and neonates are limited. Aim: This study investigated the effects of regional and seasonal variability on trace mineral status in dam serum (DS), dam milk (DM), and neonatal serum (NS) across major camel-rearing regions of Saudi Arabia. We hypothesized that environmental factors—particularly heat stress and local feed resources—drive regional and seasonal differences in mineral profiles and maternal–neonatal transfer. Methods: Samples of serum, milk, feed, water, and soil were collected from five major regions during three seasons. Concentrations of selenium (Se), zinc (Zn), copper (Cu), iron (Fe), manganese (Mn), and iodine (I) were quantified, and correlations among biological compartments were analyzed. Meteorological data were used to compute the temperature-humidity index (THI). Results: The THI ranged from thermoneutral levels in the Northern winter (17.4) to severe heat stress in Eastern summer (33.8). Milk minerals exhibited strong seasonal and regional effects, with selenium peaking in summer and zinc in spring. Western dams showed elevated iron and iodine, whereas northern dams had higher zinc. Serum minerals in dams varied moderately with season but differed regionally for zinc, selenium, and iron. Neonatal serum reflected maternal and regional influences, showing significant season-by-region interactions for selenium and iodine. Positive correlations indicated coordinated maternal–neonatal mineral transfer, particularly for selenium, iodine, and zinc. Feed represented the main environmental source of Cu and Se. In conclusion, camel trace mineral status is mainly driven by environmental factors but regulated through maternal transfer, with selenium and iodine emerging as key heat-stress markers supporting targeted, region- and season-specific supplementation to improve health and productivity in arid regions.

## 1. Introduction

The dromedary camel (*Camelus dromedarius*) is a cornerstone of food security, pastoral livelihoods, and cultural heritage in arid and semi-arid regions, especially in Saudi Arabia, which hosts the largest camel population on Arabian Peninsula [1,2,3,4]. Camels contribute significantly to meat, milk, and transport production systems and are uniquely adapted to extreme environments. Unlike intensively managed ruminants, camels graze extensively across diverse ecological zones—from hyper-arid deserts and volcanic highlands to coastal plains and mountainous regions—leading to substantial phenotypic and genetic diversity [4,5,6]. These environmental gradients shape physiological processes, including mineral metabolism and stress adaptation.

Camel milk, a key component of this system, is characterized by a distinctive biochemical composition that supports neonatal development under harsh desert conditions [7,8]. It contains balanced proportions of proteins, fats, and lactose, alongside a broad spectrum of bioactive micronutrients, including essential trace elements [9,10]. These microelements are transferred from the maternal circulation to milk primarily through active secretion mechanisms in the mammary gland involving specific metal transporters, binding proteins, and ion channels that regulate uptake and excretion rates [11,12,13]. The efficiency of this maternal transfer is influenced by diet, physiological status, and environmental stress, particularly heat load and mineral availability in feed and water resources [14,15,16].

Trace minerals such as selenium (Se), zinc (Zn), copper (Cu), iron (Fe), manganese (Mn), and iodine (I) are vital for maintaining metabolic homeostasis, immune function, antioxidant defense, reproduction, and neonatal development [11,12,13,17,18]. Selenium and zinc are integral to antioxidant and endocrine systems that safeguard reproductive tissues and embryonic development, while copper and iron are essential for hemoglobin synthesis, oxygen transport, and immune competence. Manganese supports enzymatic activity, cartilage formation, and energy metabolism, whereas iodine plays a central role in thyroid hormone synthesis and thermoregulation. Collectively, these elements form an interconnected network regulating metabolic efficiency, reproductive performance, and neonatal viability. Deficiencies or imbalances in these trace minerals can impair growth, fertility, thyroid function, and passive immunity transfer [17,18]. In livestock, maternal mineral status is a primary determinant of neonatal reserves through placental transfer and colostrum or milk intake [19,20,21]. However, most available data originate from cattle and small ruminants in temperate systems, limiting their applicability to camels under extensive desert production.

Environmental reservoirs such as soil, water, and forage are the principal mineral sources in grazing systems [22,23]. In Saudi Arabia, these sources differ markedly across regions and fluctuate seasonally [3,24]. Ongoing climate change further exacerbates rangeland degradation in arid regions, leading to reduced feed availability and declining nutritional quality [25,26,27]. Although camels are among the most climate-resilient domestic species, they remain vulnerable to extreme heat and resource scarcity [28,29]. Heat stress alters feed intake, rumen fermentation, water turnover, and metabolic partitioning, thereby affecting mineral absorption and retention [30,31,32]. The THI is widely used to quantify thermal load and has consistently been linked to reduced productivity and mineral depletion in ruminants, including camels [33,34,35,36].Recent studies have reported regional variability in camel milk and tissue mineral content, suggesting strong environmental influences on mineral metabolism [3,24]. Additionally, ref. [4] identified genomic signatures linked to desert adaptation, including genes associated with protein stability and oxidative resilience. These findings imply that mineral dynamics in camels are governed by complex interactions among environment, physiological regulation, and genetic adaptation. However, key aspects-such as seasonal influences, maternal–neonatal transfer pathways, and inter-mineral coordination-remain poorly defined.

Late gestation and early lactation are critical windows of mineral demand during which dams mobilize hepatic and skeletal reserves to support fetal development and colostral transfer [37,38]. Milk minerals are more dynamic than serum because mammary secretion reflects both circulating supply and active regulation, influenced by heat stress, milk yield, and environmental mineral availability [39]. Moreover, trace minerals interact through shared metabolic pathways-such as Se-I in thyroid function and Cu-Fe via ceruloplasmin-making their metabolism highly interdependent [11,40,41]. Despite this complexity, cross-compartmental regulation among dam serum, milk, and neonatal serum remains poorly defined in camels.

This study therefore focused on selenium, zinc, copper, iron, manganese, and iodine as the principal microelements of physiological and ecological significance in camels. These six elements were selected because of their critical roles in growth, reproduction, and stress adaptation, their environmental variability across arid regions, and their high potential for deficiency or imbalance under extensive pastoral systems typical of Saudi Arabia [1,13,42]. Accordingly, the aim of this study was to investigate regional and seasonal variation in trace minerals in dam serum, milk, and neonatal serum across major camel-rearing regions of Saudi Arabia, examined maternal–neonatal correlations, and linked these profiles to soil, water, and feed mineral availability. By adopting a systems-level approach that connects environment, dam physiology, and neonatal outcomes, this work provides novel insights into maternal transfer pathways and establishes a foundation for targeted mineral management to enhance camel productivity and resilience under increasing climatic stress and food security challenges.

## 2. Materials and Methods

### 2.1. Experimental and Regional Temperatures

The study was conducted in five semi-arid agricultural zones of Saudi Arabia: central, eastern, western, southern, and northern. All regions are characterized by a semi-arid climate with hot summers and cold winters, though the severity varies across zones [43]. The central and eastern regions typically experience the highest summer temperatures, while the northern and western regions are subject to more severe winter conditions. In contrast, the southern region, due to its higher elevation, shows a relatively moderate temperature range. Winter usually extends from mid-September to late January, with December being the coldest month. The experimental period spanned from early winter 2022 to late summer 2023 (November 2022–July 2023). The geographic distribution of the study districts is shown in Graphetically abstract.

Mean ambient temperature (AT; average between Min and max temperature), relative humidity (RH, %) between seasonal and regional variability of Saudi Arabia regions and calculated THI for the mean AT using the commonly used animal-science THI formula: THI = T − (0.55 − 0.0055 × RH) × (T − 14.5); where T = mean ambient temperature in °C, RH in %. The selected THI formula was used because it has been widely applied and validated in animal science research for quantifying thermal load under arid and semi-arid conditions. It accurately integrates both ambient temperature and relative humidity-two key environmental stressors affecting livestock in desert ecosystems and has demonstrated strong correlations with physiological and productivity responses in camels and other heat-tolerant ruminants [33,44]. Therefore, this formula is well-suited to represent the environmental conditions characteristic of Saudi Arabia’s camel-rearing regions. The mean national temperature recorded during the study was 41.6 ± 3.4 °C, with only minor variation among regions and across seasons.

### 2.2. Samples Collection

A total of 450 samples were collected, comprising 150 she-camel serum, 150 calf-serum, and 150 she-camel milk samples, each representing five regions, three seasons, and ten animals per group. Adult multiparous she-camels and their neonates (less than three months old) were sampled once per site during three seasons—summer, winter, and spring of 2022–2023. All animals were clinically healthy and managed under comparable semi-extensive grazing conditions, with offspring kept alongside their dams throughout the study period. To avoid repeated sampling from the same individuals, samples were taken from different animals within the same locale and management system. The present analyses focused solely on trace mineral concentrations in milk rather than its complete proximate composition. Therefore, interactions between macro-nutrients and mineral levels were not examined in this study.

Blood sampling was performed by licensed veterinarians using aseptic jugular venipuncture from both she-camels and their offspring across the five semi-arid regions (central, eastern, western, southern, and northern) during three seasons (summer, winter, and spring). To prepare serum, blood samples were centrifuged at 2000 rpm for 10 min. The resulting serum was treated with 10% trichloroacetic acid (TCA; 1:4, serum: TCA), followed by centrifugation at 1500× *g* for 10 min. The supernatant was carefully collected and stored at −20 °C until trace mineral determination.

Concurrently, milk samples were collected from lactating she-camels using manual milking under hygienic conditions after udder cleaning and discarding the first few streams to avoid contamination. Milk samples were processed in parallel using identical procedures for trace mineral analysis.

Feed, water, and soil sampled from the same locations at the time of biological collection. All samples were homogenized to ensure uniformity, after which representative subsamples were taken, properly labeled, and transported to the laboratory for mineral composition analysis. All samples were subsequently analyzed for mineral concentrations using inductively coupled plasma mass spectrometry (ICP–MS).

### 2.3. Mineral Analysis

Trace mineral concentrations in blood and milk samples were determined using an ICP-OES (PerkinElmer, Shelton, CT, USA) equipped with a Meinhard Type A2 nebulizer. High-purity argon (>99.99%, AH Group, Dammam, Saudi Arabia) served as plasma support and carrier gas [1]. The instrument was operated at 1300 W RF power, 15 L min^−1^ plasma flow, 0.2 L min^−1^ auxiliary flow, 0.8 L min^−1^ nebulizer flow, and 1.5 mL min^−1^ sample uptake rate. Both axial and radial viewing modes were employed, with two-point background correction and triplicate measurements. Peak area signals were recorded at interference-free, highly sensitive wavelengths. Calibration standards were prepared from a 1000 mg L^−1^ multi-element stock solution diluted in 0.5% (*v*/*v*) nitric acid, covering 1.0 ng mL^−1^ to 1.0 µg mL^−1^. Instrument accuracy was confirmed by standard solutions and recovery tests, yielding 95–102% recovery. All analyses were performed at the Animal Production Laboratories, King Saud University.

### 2.4. Statistical Analysis

Statistical analyses were conducted to evaluate the effects of region, season, and their interactions on serum and milk mineral concentrations. Data were analyzed under a completely randomized design using two-way ANOVA within the General Linear Model procedure (PROC GLM; SAS v. 9.4, SAS Institute Inc., Cary, NC, USA). The dependent variable was trace mineral concentration, while the independent variables included the main effects of season (summer, winter, spring), region (five semi-arid districts: central, eastern, western, southern, northern), and their interaction. The statistical model was specified as:

$$\gamma_{ijk}=\mu +{R_{i}+S}_{j}+{RS}_{ij}\;{+\;e}_{ijk}$$

where *γ_ijk_* represents the trace mineral concentrations (Cu, Fe, I, Mn, Se, Zn) in she-camel serum, newborn serum, and she-camel milk; *μ* is the overall mean;

*R_i_* is the effect of the *i*region; *S_j_* is the effect of the *j* season; *RS_ij_* is the interaction between region and season; and *e_ijk_* is the random error.

Model validity was assessed using coefficients of determination (R^2^) and variation coefficients (VC). Pearson correlation analysis was applied to assess relationships between trace mineral concentrations in she-camel serum and milk, and the corresponding newborn serum mineral status. Differences among means were evaluated using Tukey’s test at *p* ≤ 0.05, employing the PDIFF option of LSMEANS.

## 3. Results

### 3.1. Trace Minerals in Dam Milk

Milk trace mineral composition varied significantly with season and region (Table 1). Seasonal variations were most pronounced for Se, Zn, Cu, Fe, and I (*p* < 0.0001), whereas Mn exhibited no significant seasonal trend (*p* > 0.05). Selenium concentrations were highest in summer (4.29 µg/L), whereas zinc concentrations peaked in spring (14.34 µg/L). Regional variation was particularly evident, with Western dams exhibiting elevated iron (60.7 µg/L) and iodine (65.6 µg/L), while Northern dams showed higher Zn levels (16.53 µg/L). Season × region interactions were highly significant (*p* < 0.0001), indicating strong environmental modulation of milk trace mineral profiles.

### 3.2. Trace Minerals in Dam Serum

As shown in Table 2, serum trace mineral levels of dams were influenced by both season and region. Seasonal fluctuations were modest, though Mn concentrations were higher in spring (0.102 µg/L, *p* = 0.029). Selenium concentrations peaked in summer (1.61 µg/L), while cupremia (the concentration of Cu in the blood) tended to be higher in spring (2.69 µg/L). Regional patterns revealed elevated zincemia (the concentration of Zn in the blood) in the Western region (0.271 µg/L) and selenium in the Southern region (1.89 µg/L). Iron levels were consistently higher in the Eastern and Northern regions (*p* < 0.0001). No significant interactions (*p* > 0.05) were detected between season and region except for Cu, which was at the highest level in the Eastern regions during spring.

### 3.3. Trace Minerals in Newborn Serum

As illustrated in Table 3, the interaction between season and region had a significant effect on serum Se *(p* = 0.016) and I (*p* < 0.0001) concentrations in neonatal camels, while no interactive effects were detected for Mn, Zn, Cu, or Fe. Across main effects, seasonal differences were most pronounced for Cu (*p* = 0.03) and iodine (*p* = 0.02), with the highest mean Cu concentration observed in spring and the lowest in summer, whereas iodine was lowest in winter and peaked during spring. Although seasonal variation was not significant for Mn, Se, Zn, or Fe (*p* > 0.05), all tended to increase slightly during spring. Regional variation was significant for Se (*p* = 0.005), Zn (*p* = 0.012), Fe (*p* = 0.001), and I (*p* < 0.0001). Calves from the western region exhibited the highest Se and Zn levels, while those from the eastern region showed elevated Fe and I concentrations. Conversely, southern region values were consistently among the lowest for most minerals. These results indicate distinct spatial patterns in mineral status, reflecting regional differences in soil composition, forage quality, and environmental stressors.

### 3.4. Correlations Among Dam Serum, Dam Milk, and Their Neonatal Serum Minerals

The correlation analysis among DM, DS, and NS trace minerals revealed several significant associations (Table 4 and Table 5). As shown in Table 4, selenium concentrations in DM were moderately correlated with Zn in NS (r = 0.5, *p* = 0.04) and Fe in NS (r = 0.5, *p* = 0.04). A positive correlation was detected between iodine in DM and Se in NS (r = 0.7, *p* = 0.002). Selenium- and iodine-rich milk appeared to enhance neonatal Fe and Zn status, supporting the coordinated maternal transfer of these elements and their roles in antioxidant and thyroid function. A significant positive correlation was found between manganese and selenium concentrations in dam milk (r = 0.55, *p* = 0.03). In addition, iron in dam milk showed strong positive associations with both manganese (r = 0.73, *p* = 0.002) and selenium (r = 0.63, *p* = 0.01). Conversely, zinc displayed a significant negative correlation with selenium (r = −0.68, *p* = 0.01).

In DS, significant positive correlations were observed between Cu and both Fe (*r* = 0.8, *p* = 0.001) and iodine (*r* = 0.7, *p* = 0.01) in NS, suggesting functional interrelationships among these trace elements in systemic metabolism. Maternal serum Mn also showed a positive correlation with Zn in NS (*r* = 0.6, *p* = 0.03), reinforcing the concept of inter-element coordination in maternal–neonatal mineral balance. A notable negative correlation was observed between DS selenium and NS iron (*r* = −0.7, *p* = 0.01), suggesting an inverse association between maternal Se status and neonatal Fe concentration.

A significant negative correlation was detected between copper and selenium concentrations in dam serum (r = −0.53, *p* = 0.04). Iron in dam serum showed strong positive correlations with both manganese (r = 0.71, *p* = 0.003) and zinc (r = 0.63, *p* = 0.01). Likewise, iodine was positively correlated with selenium (r = 0.62, *p* = 0.01). In the newborn serum, zinc was positively correlated with manganese (r = 0.55, *p* = 0.03), while iodine showed a moderate positive correlation with iron (r = 0.54, *p* = 0.04).

### 3.5. Correlations Between Dam Serum and Dam Milk Trace Minerals

Cross-compartmental analysis revealed no significant associations between maternal serum and milk trace mineral concentrations (Table 6). Within maternal serum, iron showed positive correlations with Mn (*r* = 0.7, *p* = 0.003) and zinc (*r* = 0.6, *p* = 0.012). Selenium exhibited a positive correlation with iodine (*r* = 0.6, *p* = 0.01) and a negative correlation with Cu (*r* = −0.5, *p* = 0.04). In maternal milk, iron was positively correlated with Mn (*r* = 0.7, *p* = 0.001) and Se (*r* = 0.6, *p* = 0.01) while Se correlated positively with Mn (*r* = 0.6, *p* = 0.03) but negatively with zinc.

### 3.6. Environmental Sources of Trace Minerals

**Figure 1:**
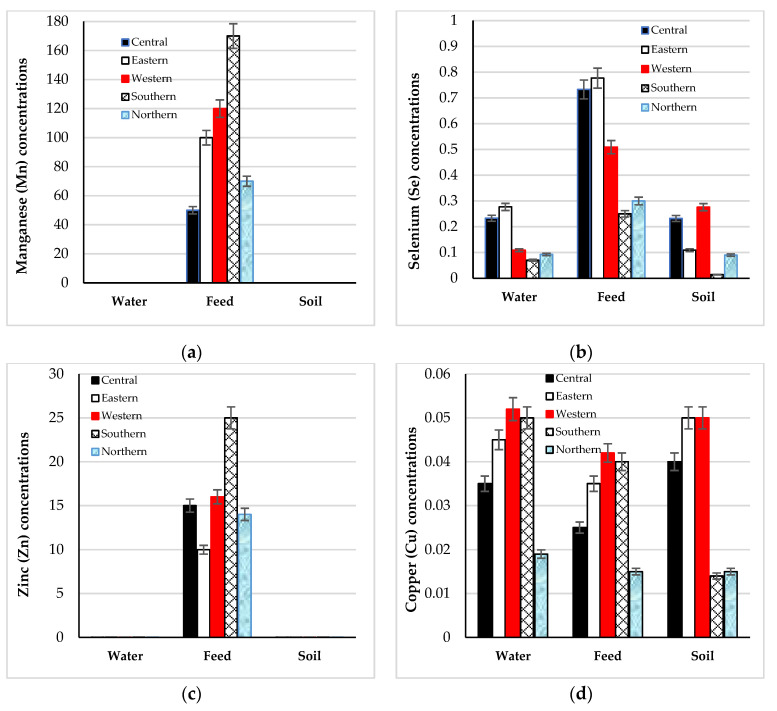

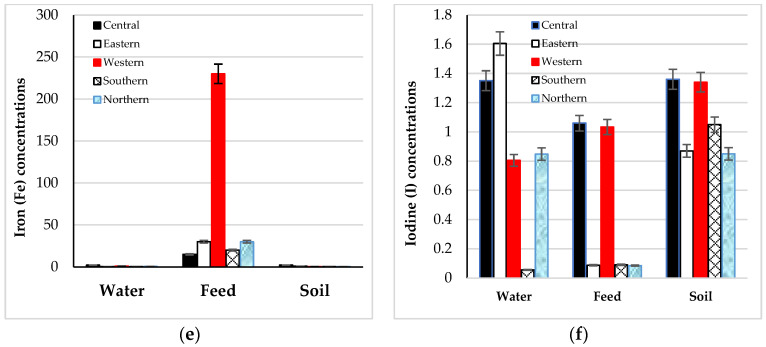
Trace mineral concentrations of (**a**) Mn, (**b**) Se, (**c**) Zn, (**d**) Cu, (**e**) Fe, and (**f**) iodine in drinking water (µg/mL), soil (ppb), and feed (µg/g) in five Saudi Arabia regions.

Water, soil, and feed samples collected from different regions exhibited considerable variation in trace element concentrations (Figure 1a–f). Manganese (Figure 1a), selenium (Figure 1b), zinc (Figure 1c), copper (Figure 1d), iron (Figure 1e), and iodine (Figure 1f) followed a similar trend, with feed serving as the primary source of these elements compared with water and soil. In feed, the Southern region recorded the highest Mn (170 µg/g) and Zn (25 µg/g) concentrations, whereas the Western region exhibited the greatest Fe concentration (230 µg/g). Copper levels were also highest in the Western region across feed, soil, and water samples. Iodine concentrations were greatest in feed and soil from the Western and Central regions, while water samples from the Eastern and Central regions showed the highest iodine content.

### 3.7. Environmental Conditions

**Figure 2:**
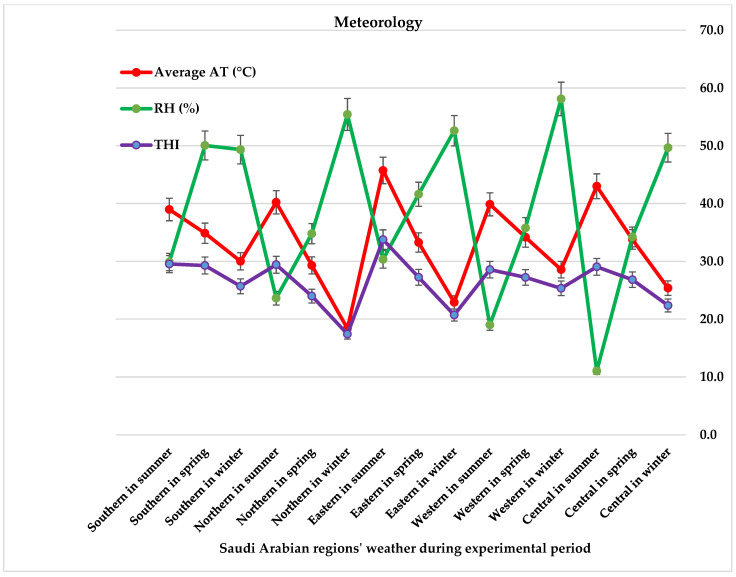
Minimum and maximum temperatures (°C), mean ambient temperature (AT, °C), relative humidity (RH, %), and temperature-humidity index (THI) for five regions Central, Eastern, Western, Southern and Northern of Saudi Arabia across spring, winter, and summer from General Authority for Statistics (GAS, 2019) and National Centre for Meteorology sources.

Meteorological records indicated pronounced seasonal and regional variation in thermal load across the five Saudi regions during the experimental period (Figure 2). Maximum air temperatures (AT) were consistently highest during summer, particularly in the Southern and Eastern regions, where values exceeded 40 °C. In contrast, winter minima dropped sharply in the Northern and Central regions. Mean AT followed similar patterns, with spring presenting intermediate values across all regions. The highest mean AT was recorded in the Eastern region during summer (45.7 °C), while the lowest occurred in the Northern region during winter (18.4 °C). The Central region also experienced pronounced seasonal shifts, with mean AT ranging from 25.4 °C in winter to 43.0 °C in summer. Relative humidity (RH) peaked in the Western coastal region during winter (58.1%), in the Northern region during winter (55.4%), and in the Southern region during spring (50.1%), whereas the driest conditions were observed in the Central region in summer (11.0%). Consequently, these climatic extremes were reflected in the THI, which ranged from cold stress /thermoneutral in the Northern winter (17.4) to heat-stress conditions in the Eastern summer (33.8). Intermediate THI values were observed in most regions during spring.

## 4. Discussion

Regional and seasonal variation in maternal and neonatal mineral profiles was primarily influenced by climatic conditions and environmental conditions, particularly the THI, which reflected the severity of heat stress across Saudi regions. Across Saudi Arabia, the THI ranged from thermoneutral in the Northern winter to severe heat stress in the Eastern and Southern summer, creating a pronounced thermal gradient. Elevated THI during summer imposed thermal and nutritional stress that can suppress feed intake, impair rumen fermentation, and alter hepatic mineral storage, thereby reducing elements such as Cu and Zn. These findings agree with previous reports demonstrating heat induced alterations in mineral metabolism and antioxidant activity in camels and other ruminants [33,34,35]. Conversely, cooler winter conditions supported more stable mineral homeostasis, confirming that ambient temperature and humidity strongly influence mineral dynamics. These findings are consistent with previous research indicating that camel mineral status is strongly influenced by environmental variability and climatic stress [3,4,11,45].

Environmental reservoirs, including soil, water, and forage, further shaped regional differences in mineral status. Western and eastern soils were rich in Fe and Zn, whereas the northern region contained higher Iodine concentrations. Because camel feed originates primarily from native forage, regional soil composition directly affects mineral intake. The observation that feed contained more Cu and Se than soil or water indicates that forages act as the main pathway linking environmental mineral availability to physiological status. This ecosystem dependence aligns with prior findings in arid livestock systems [46,47,48], emphasizing the need for continuous monitoring and supplementation programs tailored to regional mineral imbalances.

The variability in milk mineral composition compared with serum indicates that mammary secretion is highly responsive to both maternal physiological status and environmental exposure. Elevated iron and iodine concentrations in milk from western dams may reflect mineral-rich soil and water or iodophore use, consistent with geographic variation previously reported in camel milk [3]. While seasonal peaks in Se and Zn suggest that mammary regulation adjusts secretion rates in response to changing dietary availability and metabolic demand. These patterns highlight the camel’s adaptive mechanisms to maintain neonatal mineral supply under environmental stress. The high reactivity of milk minerals such as Se and Zn also implies their importance in neonatal antioxidant defense and immune development [49,50].

The relative stability of maternal serum mineral levels compared with milk variability suggests efficient homeostatic regulation through hepatic storage, skeletal mobilization, and selective absorption. Increased serum Se and Cu during warmer months likely supports enhanced antioxidant and enzymatic functions needed to counter oxidative stress [51]. Regional differences in Zn, Fe, and Se corresponded to soil and feed mineral concentrations, reflecting the camel’s capacity to regulate plasma levels while still being constrained by dietary input. Maternal serum mineral values generally remained within reference ranges for camels [11], reflecting effective internal regulation despite external stressors.

Neonatal serum profiles closely mirrored maternal and environmental trends, demonstrating coordinated mineral transfer from dam to offspring. Higher Se and Zn levels in neonates from western and eastern regions and the significant season × region interactions for Se and iodine reflects both the maternal status and the quality of colostrum and milk. Positive correlations between milk Se and neonatal Fe and Zn indicate enhanced neonatal uptake, likely mediated by antioxidant protection and increased transporter activity, while associations between milk iodine and neonatal Se support synergistic regulation of thyroid and redox pathways. Similar maternal- neonatal mineral relationships have been observed in cattle and small ruminants, confirming that adequate maternal supplementation improves neonatal reserves [52,53].

Physiologically, these maternal–offspring relationships emphasize two complementary pathways of mineral transfer: systemic transport through the dam’s circulation and selective secretion into milk. Selenium in milk exhibited moderate positive correlations with neonatal Zn and iodine, suggesting that Se-rich milk may facilitate intestinal absorption and metabolic utilization of other trace minerals by maintaining epithelial integrity, and oxidative balance. The strong correlation between milk iodine and neonatal Se supports an integrated endocrine–antioxidant mechanism, where iodine-dependent thyroid hormone activity interacts with Se-dependent deiodinase enzymes to maintain thermoregulation and postnatal adaptation.

Within maternal serum, coordinated regulation and mineral antagonisms were evident. Negative correlation between maternal Se with neonatal Cu suggests competition during intestinal absorption or hepatic storage, whereas positive relationships among Fe, Mn, and Zn reflect shared enzymatic functions in oxidative metabolism and erythropoiesis. The positive association between Se and I is physiologically relevant, as Se-dependent enzymes activate thyroid hormones, linking antioxidant protection to metabolic rate. These findings collectively underscore the interconnectedness of mineral metabolism, where the imbalance of one element can influence the function or transport of others.

The mammary gland also demonstrated selective regulatory control. Positive correlations between milk Se and Mn suggest synchronized secretion of antioxidant cofactors that protect neonatal tissues. In contrast, the inverse association between Zn and Se may indicate differential binding to milk proteins or competitive transport across mammary epithelium. Iron’s positive correlation with both Mn and Se reinforces their combined roles in neonatal oxygen transport, antioxidant protection, and enzymatic maturation. Such relationships highlight the need to view mineral nutrition as an integrated system rather than as independent elements.

From a nutritional standpoint, these physiological and metabolic insights emphasize the necessity of region-targeted supplementation strategies. Mineral mixtures enriched with Se, Zn, Cu, Fe, and I should be adjusted seasonally, particularly during periods of high THI, when mineral mobilization and oxidative stress are most pronounced. Supplementation through fortified feed, mineral blocks, or controlled oral dosing could enhance maternal reserves, improve colostrum and milk quality, and ultimately strengthen neonatal vitality and thermotolerance. Integrating mineral monitoring with soil and forage analysis would enable precision nutrition programs adapted to regional deficiencies and climatic variability.

Overall, this study demonstrates that camel mineral metabolism is shaped by the interplay between environmental exposure, physiological regulation, and nutritional adaptation. The coordinated maternal–neonatal mineral transfer system underscores the camel’s resilience to heat and nutrient stress, yet also its vulnerability to prolonged mineral imbalance under changing climatic conditions. A limitation of this study is that the analyses were restricted to trace mineral concentrations rather than the complete proximate composition of camel milk. Consequently, potential associations between macro-nutrient content (e.g., protein, fat, lactose) and mineral metabolism could not be assessed. Future studies combining proximate and mineral composition analyses would offer deeper insight into nutritional physiology and maternal–neonatal nutrient transfer mechanisms in camels under variable environmental stressors.

## 5. Conclusions

This study provides new evidence that the trace mineral status of dromedary camels is regulated through a dynamic interplay between environmental exposure, dietary mineral variability, and physiological homeostasis. Despite pronounced regional and seasonal variability in climate, lactating dams were able to maintain relatively stable serum mineral concentrations, reflecting strong internal regulatory mechanisms. In contrast, milk mineral composition exhibited marked environmental sensitivity, directly influencing neonatal serum mineral profiles. The consistent positive correlations between maternal and neonatal mineral levels indicated that maternal transfer serves as the primary pathway governing neonatal mineral supply. Selenium and iodine emerged as key indicators of climatic adaptation and oxidative-endocrine balance, while zinc, copper, and iron were closely associated with metabolic and hematopoietic stability. Regional variations in soil, water, and feed composition further revealed the ecological basis of mineral disparities among production zones in Saudi Arabia. Collectively, these findings advance the understanding of mineral metabolism in camels and emphasize the species’ remarkable adaptive capacity to heat-stress environments, while also exposing its vulnerability to prolonged mineral imbalance under extreme conditions. From a practical perspective, the results support the implementation of region-specific mineral surveillance and seasonally adjusted supplementation programs that prioritize selenium, iodine, zinc, copper, and iron. Integrating climatic data with feed and soil analysis can improve nutritional precision, enhance reproductive efficiency and sustain milk quality, and neonatal health in arid and semi-arid production systems.

## Figures and Tables

**Table 1:** Mean values of trace minerals (µg/L) in dam milk according to seasons and district and their interactions.

Treatment Arrangement		Trace Minerals in Dam Milk (µg/L)					
Season Level	Region Level	Mn	Se	Zn	Cu	Fe	I
Season × Region Interaction							
1	C	1.98 ^b^	4.11 ^bcd^	2.52 ^c^	7.41 ^bcde^	24.21 ^bc^	56.49 ^b^
1	E	31.89 ^ab^	6.64 ^a^	2.43 ^c^	15.04 ^a^	28.89 ^b^	9.54 ^cd^
1	W	1.72 ^b^	5.32 ^b^	2.49 ^c^	4.20 ^def^	29.59 ^b^	16.93 ^cd^
1	S	40.36 ^ab^	2.86 ^de^	2.54 ^c^	5.15 ^def^	22.52 ^bc^	20.90 ^cd^
1	N	2.26 ^b^	2.54 ^ef^	2.31 ^c^	6.64 ^bcdef^	29.92 ^b^	9.09 ^cd^
2	C	2.37 ^b^	3.50 ^de^	1.96 ^c^	2.44 ^ef^	26.97 ^b^	33.21 ^bc^
2	E	1.58 ^b^	3.47 ^de^	1.59 ^c^	2.18 ^f^	24.02 ^bc^	20.91 ^cd^
2	W	48.74 ^a^	6.70 ^a^	2.51 ^c^	10.04 ^abc^	139.49 ^a^	16.03 ^cd^
2	S	5.27 ^b^	2.67 ^e^	3.40 ^c^	3.21 ^ef^	32.19 ^b^	3.27 ^d^
2	N	1.67 ^b^	0.61 ^g^	18.63 ^b^	7.44 ^bcde^	5.80 ^d^	0.92 ^d^
3	C	2.20 ^b^	3.55 ^de^	3.55 ^c^	10.30 ^abc^	23.88 ^bc^	29.39 ^c^
3	E	1.99 ^b^	5.14 ^bc^	4.33 ^c^	4.28 ^def^	31.20 ^b^	23.13 ^cd^
3	W	1.92 ^b^	3.83 ^cde^	4.24 ^c^	2.09 ^f^	13.00 ^cd^	163.77 ^a^
3	S	2.04 ^b^	1.29 ^fg^	30.92 ^a^	8.85 ^bcd^	11.44 ^cd^	0.95 ^d^
3	N	1.78 ^b^	1.05 ^g^	28.66 ^a^	11.24 ^ab^	6.52 ^d^	1.49 ^d^
SE		3.671	0.104	0.277	0.401	1.029	2.015
Season							
Summer		15.64	4.29 ^a^	2.46 ^c^	7.69 ^a^	27.03 ^b^	22.49 ^b^
Winter		11.92	3.39 ^b^	5.62 ^b^	5.06 ^b^	45.69 ^a^	14.87 ^c^
Spring		1.99	2.97 ^c^	14.34 ^a^	7.35 ^a^	17.21 ^c^	43.74 ^a^
SE		4.74	0.134	0.357	0.517	1.33	2.60
Region							
C		2.18	3.72 ^b^	2.68 ^c^	6.72 ^bc^	25.01 ^b^	39.70 ^b^
E		11.82	5.08 ^a^	2.78 ^c^	7.17 ^ab^	28.04 ^b^	17.86 ^c^
W		17.46	5.28 ^a^	3.08 ^c^	5.45 ^c^	60.70 ^a^	65.58 ^a^
S		15.89	2.27 ^c^	12.29 ^b^	5.95 ^bc^	22.05 ^b^	8.12 ^cd^
N		1.91	1.40 ^d^	16.53 ^a^	8.44 ^a^	14.08 ^c^	3.83 ^d^
SE		8.21	0.233	0.619	0.896	2.30	4.51
*p*-value							
Season		0.138	<0.0001	<0.0001	<0.0001	<0.0001	<0.0001
Region		0.173	<0.0001	<0.0001	0.008	<0.0001	<0.0001
Season × Region interaction		0.009	<0.0001	<0.0001	<0.0001	<0.0001	<0.0001

Regions: C: Central, E: Eastern, W: Western, S: Southern and N: Northern. Seasons: 1 = Summer, 2 = Winter, 3 = Spring. ^a–g^ Within a column, means without a common superscript are significantly different. SE: Standard error; *p* ≤ 0.05 is considered to be significantly different.

**Table 2:** Mean values of trace minerals (µg/L) in dam serum according to seasons and district and their interactions.

Treatment Arrangement		Trace Minerals in Dam Serum (µg/L)					
Season Level	Region Level	Mn	Se	Zn	Cu	Fe	I
Season × Region interaction							
1	C	0.073	1.86	0.121	1.15 ^b^	0.472	0.112
1	E	0.096	1.19	0.218	2.24 ^ab^	0.834	0.150
1	W	0.095	1.61	0.307	2.49 ^ab^	0.674	0.184
1	S	0.084	1.93	0.229	1.86 ^b^	0.688	0.225
1	N	0.087	1.59	0.275	2.71 ^ab^	0.753	0.208
2	C	0.083	1.11	0.143	1.95 ^b^	0.560	0.072
2	E	0.087	1.17	0.183	2.03 ^b^	0.630	0.166
2	W	0.099	1.25	0.234	1.75 ^b^	0.717	0.190
2	S	0.081	1.75	0.169	1.39 ^b^	0.579	0.212
2	N	0.103	1.51	0.264	2.04 ^b^	0.720	0.264
3	C	0.087	1.03	0.154	3.02 ^ab^	0.557	0.088
3	E	0.106	1.14	0.199	4.50 ^a^	0.745	0.148
3	W	0.121	1.37	0.188	2.01 ^b^	0.760	0.185
3	S	0.097	1.94	0.167	1.55 ^b^	0.614	0.275
3	N	0.105	1.32	0.211	2.28 ^ab^	0.818	0.161
SE		0.008	0.176	0.029	0.414	0.054	0.051
Season							
Summer		0.088 ^b^	1.61 ^a^	0.236 ^a^	2.14 ^ab^	0.683	0.172
Winter		0.091 ^ab^	1.29 ^b^	0.198 ^ab^	1.91 ^b^	0.643	0.170
Spring		0.102 ^a^	1.34 ^ab^	0.182 ^b^	2.69 ^a^	0.690	0.166
SE		0.003	0.079	0.013	0.185	0.024	0.023
Region							
C		0.079 ^b^	1.41 ^b^	0.135 ^c^	1.080 ^ab^	0.523 ^b^	0.092
E		0.094 ^ab^	1.17 ^c^	0.201 ^bc^	2.46 ^a^	0.738 ^a^	0.156
W		0.099 ^a^	1.48 ^b^	0.271 ^a^	2.21 ^ab^	0.697 ^a^	0.186
S		0.087 ^ab^	1.89 ^a^	0.198 ^bc^	1.671 ^b^	0.642 ^ab^	0.234
N		0.096 ^ab^	1.52 ^b^	0.261 ^ab^	2.39 ^ab^	0.750 ^a^	0.222
SE		0.004	0.102	0.017	0.239	0.031	0.029
*p*-value							
Season		0.029	0.019	0.064	0.051	0.321	0.971
Region		0.007	0.005	0.0002	0.031	<0.0001	0.028
Season × Region interaction		0.707	0.283	0.413	0.029	0.117	0.969

Regions: C: Central, E: Eastern, W: Western, S: Southern and N: Northern. Seasons: 1 = Summer, 2 = Winter, 3 = Spring. ^a–c^ Within a column, means without a common superscript are significantly different. SE: Standard error; *p*-value ≤ 0.05 is considered to be significantly different.

**Table 3:** Mean values of trace minerals (µg/L) in neonatal serum according to seasons and district and their interactions.

Treatment Arrangement		Trace Minerals in Neonatal Serum (µg/L)					
Season Level	Region Level	Mn	Se	Zn	Cu	Fe	I
Seasons × Region Interaction							
1	C	0.074	2.12 ^ab^	0.171	0.752	0.512	0.341 ^b^
1	E	0.102	1.19 ^c^	0.230	2.332	0.798	0.105 ^c^
1	W	0.165	1.72 ^abc^	0.325	2.435	0.761	0.277 ^bc^
1	S	0.087	1.22 ^c^	0.214	2.118	0.694	0.107 ^c^
1	N	0.10	1.17 ^c^	0.172b	1.224	0.614	0.614 ^a^
2	C	0.086	1.23 ^c^	0.193	2.138	0.657	0.118 ^bc^
2	E	0.109	1.26 ^c^	0.207	2.998	0.798	0.299 ^bc^
2	W	0.095	1.79 ^abc^	0.256	1.824	0.694	0.161 ^bc^
2	S	0.105	1.34 ^bc^	0.178	2.773	0.579	0.080 ^c^
2	N	0.084	1.45 ^bc^	0.191	1.995	0.573	0.170 ^bc^
3	C	0.115	1.55 ^bc^	0.192	2.836	0.832	0.197 ^bc^
3	E	0.130	1.73 ^abc^	0.232	2.644	0.938	0.833 ^a^
3	W	0.123	2.44 ^a^	0.302	2.300	0.705	0.275 ^bc^
3	S	0.125	1.49 ^bc^	0.176	3.106	0.613	0.104 ^c^
3	N	0.149	1.19 ^c^	0.190	2.273	0.754	0.094 ^c^
SE		0.031	0.208	0.037	0.415	0.059	0.061
Seasons							
Summer		0.106	1.48	0.235	1.77 ^b^	0.676	0.289 ^ab^
Winter		0.096	1.41	0.205	2.35 ^ab^	0.660	0.166 ^b^
Spring		0.128	1.68	0.218	2.63 ^a^	0.768	0.301 ^a^
SE		0.014	0.093	0.017	0.185	0.027	0.027
Region							
C		0.092	1.63 ^ab^	0.185 ^b^	1.91	0.667 ^b^	0.219 ^ab^
E		0.114	1.39 ^b^	0.223 ^ab^	2.66	0.845 ^a^	0.412 ^a^
W		0.128	1.99 ^a^	0.294 ^a^	2.19	0.720 ^ab^	0.238 ^a^
S		0.106	1.35 ^b^	0.189 ^b^	2.67	0.629 ^b^	0.097 ^b^
N		0.111	1.27 ^ab^	0.191 ^b^	1.83	0.647 ^b^	0.293 ^ab^
SE		0.018	0.120	0.022	0.239	0.034	0.035
*p*-value							
Season		0.419	0.282	0.602	0.031	0.066	0.017
Region		0.744	0.005	0.012	0.142	0.001	<0.0001
Season × Region interaction		0.835	0.016	0.932	0.117	0.056	<0.0001

Regions: C: Central, E: Eastern, W: Western, S: Southern and N: Northern. Seasons: 1 = Summer, 2 = Winter, 3 = Spring. ^a–c^ Within a column, means without a common superscript are significantly different. SE: Standard error. *p*-value ≤ 0.05 is considered to be significantly different.

**Table 4:** Correlation coefficients matrix between trace minerals, µg/L concentrations in milk of dams with their newborn serum.

Pearson Correlation Coefficients, N = 150												
Prob > |r| Under H0: Rho = 0												
Minerals	DM^Mn^	DM^Se^	DM^Zn^	DM^Cu^	DM^Fe^	DM^I^	NS^Mn^	NS^Se^	NS^Zn^	NS^Cu^	NS^Fe^	NS^I^
DM^Mn^	1											
										
DM^Se^	0.55	1										
	0.03											
DM^Zn^	−0.26	−0.68	1									
	0.35	0.01										
DM^Cu^	0.44	0.13	0.34	1								
	0.10	0.64	0.21									
DM^Fe^	0.73	0.63	−0.35	0.17	1							
	0.002	0.01	0.20	0.55								
DM^I^	−0.15	0.18	−0.28	−0.39	−0.11	1						
	0.59	0.53	0.31	0.15	0.70							
NS^Mn^	−0.29	0.18	0.13	−0.14	−0.11	0.07	1					
	0.30	0.53	0.65	0.62	0.69	0.81						
NS^Se^	0.02	0.38	−0.15	−0.17	0.31	0.72	0.22	1				
	0.93	0.17	0.59	0.55	0.26	0.002	0.44					
NS^Zn^	0.20	0.54	−0.31	−0.23	0.24	0.45	0.55	0.48	1			
	0.47	0.04	0.26	0.42	0.39	0.09	0.03	0.07				
NS^Cu^	−0.17	−0.26	0.42	−0.09	−0.21	−0.10	0.48	−0.06	0.02	1		
	0.53	0.36	0.12	0.76	0.45	0.72	0.07	0.84	0.93			
NS^Fe^	0.11	0.53	−0.32	0.00	0.10	0.04	0.52	−0.01	0.42	0.19	1	
	0.70	0.04	0.24	0.99	0.73	0.88	0.05	0.97	0.12	0.49		
NS^I^	−0.31	0.33	−0.25	−0.31	−0.01	0.27	0.36	0.41	0.23	−0.19	0.54	1
	0.27	0.22	0.36	0.26	0.97	0.34	0.18	0.13	0.41	0.50	0.04	

DM and NS denote dam milk and newborn serum, respectively. Dark green text with green fill for values of r that are noticeably positive and dark red text with light red fill for values of r that are noticeably negative. Dark yellow text with yellow fill for *p*-values less than 0.05. Each cell presents two values: the correlation coefficient (r) and its corresponding *p*-value, respectively.

**Table 5:** Correlation coefficients matrix between trace minerals, µg/L concentrations in serum of dams with their newborn serum.

Pearson Correlation Coefficients, N = 150												
Prob > |r| Under H0: Rho = 0												
Minerals	DS^Mn^	DS^Se^	DS^Zn^	DS^Cu^	DS^Fe^	DS^I^	NS^Mn^	NS^Se^	NS^Zn^	NS^Cu^	NS^Fe^	NS^I^
DS^Mn^	1											
										
DS^Se^	−0.30	1										
	0.28											
DS^Zn^	0.33	0.09	1									
	0.23	0.76										
DS^Cu^	0.36	−0.53	0.25	1								
	0.19	0.04	0.37									
DS^Fe^	0.71	−0.26	0.63	0.38	1							
	0.003	0.36	0.01	0.16								
DS^I^	0.29	0.62	0.50	−0.24	0.31	1						
	0.30	0.01	0.06	0.38	0.25							
NS^Mn^	0.44	−0.10	0.33	0.45	0.25	0.07	1					
	0.10	0.71	0.23	0.10	0.37	0.80						
NS^Se^	0.37	0.03	−0.22	−0.09	−0.12	−0.13	0.22	1				
	0.18	0.92	0.44	0.74	0.68	0.63	0.44					
NS^Zn^	0.56	−0.17	0.50	0.19	0.35	0.07	0.55	0.48	1			
	0.03	0.54	0.06	0.50	0.20	0.79	0.03	0.07				
NS^Cu^	0.28	0.09	−0.15	0.03	−0.02	0.39	0.48	−0.06	0.02	1		
	0.31	0.76	0.58	0.92	0.96	0.15	0.07	0.84	0.93			
NS^Fe^	0.32	−0.65	0.11	0.78	0.31	−0.33	0.52	−0.01	0.42	0.19	1	
	0.24	0.01	0.69	0.00	0.25	0.23	0.05	0.97	0.12	0.49		
NS^I^	0.21	−0.26	−0.07	0.68	0.03	−0.32	0.36	0.41	0.23	−0.19	0.54	1
	0.46	0.35	0.82	0.01	0.90	0.24	0.18	0.13	0.41	0.50	0.04	

DS and NS denote dam serum and newborn serum, respectively. Dark green text with green fill for values of r that are noticeably positive and dark red text with light red fill for values of r that are noticeably negative. Dark yellow text with yellow fill for *p*-values less than 0.05. Each cell presents two values: the correlation coefficient (r) and its corresponding *p*-value, respectively.

**Table 6:** Correlation coefficients matrix between trace minerals, µg/L concentrations in serum and milk of camel’s dam.

Minerals	DS^Mn^	DS^Se^	DS^Zn^	DS^Cu^	DS^Fe^	DS^I^	DM^Mn^	DM^Se^	DM^Zn^	DM^Cu^	DM^Fe^	DM^I^
DS^Mn^	1											
										
DS^Se^	−0.30	1										
	0.28											
DS^Zn^	0.33	0.09	1									
	0.23	0.76										
DS^Cu^	0.36	−0.53	0.25	1								
	0.19	0.04	0.37									
DS^Fe^	0.71	−0.26	0.63	0.38	1							
	0.003	0.36	0.01	0.16								
DS^I^	0.29	0.62	0.50	−0.24	0.31	1						
	0.30	0.01	0.06	0.38	0.25							
DM^Mn^	−0.01	−0.02	0.22	−0.19	0.31	0.12	1					
	0.97	0.94	0.43	0.50	0.27	0.68						
DM^Se^	0.01	−0.38	0.07	0.21	0.11	−0.42	0.55	1				
	0.96	0.16	0.80	0.44	0.69	0.11	0.03					
DM^Zn^	0.36	0.25	0.00	−0.13	0.20	0.47	−0.26	−0.68	1			
	0.19	0.36	1.00	0.64	0.48	0.08	0.35	0.01				
DM^Cu^	0.05	−0.09	0.09	−0.01	0.34	0.00	0.44	0.13	0.34	1		
	0.85	0.74	0.75	0.97	0.21	1.00	0.10	0.64	0.21			
DM^Fe^	−0.03	−0.20	0.15	−0.07	0.04	−0.05	0.73	0.63	−0.35	0.17	1	
	0.92	0.47	0.60	0.80	0.88	0.86	0.001	0.01	0.20	0.55		
DM^I^	0.40	−0.10	−0.27	−0.07	−0.02	−0.22	−0.15	0.18	−0.28	−0.39	−0.11	1
	0.138	0.72	0.33	0.81	0.95	0.42	0.59	0.53	0.31	0.15	0.70	

DS, DM and NS denote dam serum, dam milk and newborn serum, respectively. Dark green text with green fill for values of r that are noticeably positive and dark red text with light red fill for values of r that are noticeably negative. Dark yellow text with yellow fill for *p*-values less than 0.05. Each cell presents two values: the correlation coefficient (r) and its corresponding *p*-value, respectively.

## Data Availability

The original contributions presented in the study are included in the article; further inquiries can be directed to the corresponding authors.

## References

[1] Abdelrahman M.M., Alhidary I.A., Alobre M.M., Matar A.M., Al-Badwi M.A., Qaid M.M., Aljumaah R.S. (2025). Trace elements levels in growing camels’(*Camelus dromedarius*) biological tissues from semi-arid areas. J. Appl. Anim. Res..

[2] Ibrahim M.S.I., Hafez A.-E.E., El Bayomi R.M., Mahmoud A.F.A. (2025). Review on Camel Meat: Health Benefits, Chemical Contaminants, Health Risks, and Mitigation Strategies. Egypt. J. Vet. Sci..

[3] Konuspayeva G., Faye B., Bengoumi M. (2022). Mineral status in camel milk: A critical review. Anim. Front..

[4] Ibrahim M.A., Tolone M., Barbato M., Alsubaie F.M., Alrefaei A.F., Almutairi M. (2025). Geographical distribution, genetic diversity, and environmental adaptations of dromedary camel breeds in Saudi Arabia. Front. Vet. Sci..

[5] Boukrouh S., Ait El Alia O., Faye B. (2025). Worldwide camel meat and products: An extensive analysis of production, consumption patterns, market evolution, and supply chain effectiveness. Meat Sci..

[6] Mohammed A., Almuyidi A., Almarri H., Alkhalifah H., Alhmad A., Alali H., AlHuwaish O., AlKhawaher M. (2025). Unique Characteristics of Camel Body Systems: Adaptation to Harsh Conditions, Productive and Reproductive Performances: A Review. Indian. J. Anim. Res.

[7] Faustini M., Vigo D., Brecchia G., Agradi S., Draghi S., Curone G., Atigui M., Sboui A., Quattrone A., Fehri N.E. (2025). Camel (*Camelus dromedarius* L. and *Camelus bactrianus* L.) Milk Composition and Effects on Human Type 1 and Type 2 Diabetes Mellitus: A Review. Biology.

[8] Tsognemekh B., Sumiya G., Shinji T., Motohiro H., Purevdorj N.-O. (2025). Seasonal changes in the yield and composition of camel milk in Mongolia. Pastor. Res. Policy Pract..

[9] Voronina O., Bogolyubova N., Zaitsev S.Y. (2022). Mineral composition of cow milk—A mini review. Sel’skokhozyaystvennaya Biol..

[10] Muthukumaran M.S., Mudgil P., Baba W.N., Ayoub M.A., Maqsood S. (2023). A comprehensive review on health benefits, nutritional composition and processed products of camel milk. Food Rev. Int..

[11] Abdelrahman M.M., Alhidary I.A., Aljumaah R.S., Faye B. (2022). Blood trace element status in camels: A review. Animals.

[12] El-Sayed A., Ebissy E., Mohamed R., Ateya A. (2024). Effects of antioxidant vitamins (A, D, E) and trace elements (Cu, Mn, Se, Zn) administration on gene expression, metabolic, antioxidants and immunological profiles during transition period in dromedary camels. BMC Vet. Res..

[13] Kumar R., Singh K.D., Chauhan S.S., Verma M.K., Verma A.K., Singh J., Srivastva S., Kumar P. (2025). Trace Minerals in Growth, Production and Reproduction in Farm Animals. Indian J. Anim. Reprod..

[14] Kebir N.E., Berber N., Zahzeh M.R. (2024). Anatomical and physiological properties of the dromedary: A potential sustainability alternative and a vital asset in the era of climate change. J. Anim. Behav. Biometeorol..

[15] Al Jassim R. (2024). Good feeding: Nutrition and feeding of the Arabian camel (*Camelus dromedarius*). Dromedary Camel Behavior and Welfare: Camel Friendly Management Practices.

[16] Ali M.A., Abu Damir H., Adem M.A., Ali O.M., Amir N., Shah A.A., Al Muhairi S.S., Al Abdouli K.O., Khawaja J.R., Fagieri T.A. (2023). Effects of long-term dehydration on stress markers, blood parameters, and tissue morphology in the dromedary camel (*Camelus dromedarius*). Front. Vet. Sci..

[17] Wróblewski M., Wróblewska W., Sobiesiak M. (2024). The role of selected elements in oxidative stress protection: Key to healthy fertility and reproduction. Int. J. Mol. Sci..

[18] Kumar M., Kumar D., Sharma A., Bhadauria S., Thakur A., Bhatia A. (2024). Micronutrients throughout the life cycle: Needs and functions in health and disease. Curr. Nutr. Food Sci..

[19] Silva F.G., Silva S.R., Pereira A.M., Cerqueira J.L., Conceição C. (2024). A comprehensive review of bovine colostrum components and selected aspects regarding their impact on neonatal calf physiology. Animals.

[20] Jenkins A.K. (2022). Biological and Management Factors Affecting Colostrum Intake and Pre-Weaning Success in Piglets.

[21] Van Emon M., Sanford C., McCoski S. (2020). Impacts of bovine trace mineral supplementation on maternal and offspring production and health. Animals.

[22] Askar A.R., Masoud A., El-Bordeny N.E., Kewan K.Z., El-Galil E.R.A., El Ezz S.S.A., Shoukry M.M. (2024). Grazing camels under semi-extensive production system: Selectivity, feed intake capacity, digestion and energy expenditure. BMC Vet. Res..

[23] Duguma B., Janssens G.P. (2021). Assessment of livestock feed resources and coping strategies with dry season feed scarcity in mixed crop–livestock farming systems around the gilgel gibe catchment, Southwest Ethiopia. Sustainability.

[24] Abdelrahman M.M., Qaid M.M., Al-Badwi M.A., Alobre M.M., Matar A.M., Aljumaah R.S., Alhidary I.A. (2025). Calcium, Phosphorus and Magnesium Status: A Correlation between Camelids and Their Newborns in Semi-arid District. Indian J. Anim. Res..

[25] Steinfeld H., Gerber P., Wassenaar T.D., Castel V., De Haan C. (2006). Livestock’s Long Shadow: Environmental Issues and Options.

[26] Mugoti A., Nyamukanza C., Munengwa A., Moyo S., Chikumba N. (2025). Effects of climate change on goat production and mitigatory measures in semiarid savanna ecosystems. J. Integr. Environ. Sci..

[27] Nazir N. (2025). Estimating the Nexus between Climate Change and Livestock Production in Pakistan. J. Econ. Sci..

[28] Burger P.A., Ciani E., Faye B. (2019). Old World camels in a modern world—A balancing act between conservation and genetic improvement. Anim. Genet..

[29] Abdallah H., Faye B. (2013). Typology of camel farming system in Saudi Arabia. Emir. J. Food Agric..

[30] Samara E.M., Al-Badwi M.A., Abdoun K.A., Abdelrahman M.M., Okab A.B., Bahadi M.A., Al-Haidary A.A. (2025). The interrelationship between macrominerals and heat stress in ruminants: Current perspectives and future directions—A review. Vet. Res. Commun..

[31] Muzzo B.I., Ramsey R.D., Villalba J.J. (2024). Changes in climate and their implications for cattle nutrition and management. Climate.

[32] Neves L.F., Gomes M.B., Campolina J.P., Campos M.M., Souza E.M., Diavão J., Silva A.S., Tomich T.R., Carvalho W.A., Lage H.F. (2025). Impact of Heat Stress on Intake, Performance, Digestibility, and Health of Neonatal Dairy Calves. Animals.

[33] Omran F.I. (2024). Climatic challenges for domestic ruminants under the Egyptian conditions in relation to temperature humidity index (THI). Egypt. J. Agric. Res..

[34] Faraz A., Masebo N.T., Hussain S.M., Waheed A., Ishaq H.M., Tauqir N.A., Abbasi A.R., Saleem F., Padalino B. (2025). Association of environmental temperature and relative humidity with ocular and flank temperatures in dromedary camels. Animals.

[35] Habte M., Eshetu M., Maryo M., Andualem D., Legesse A., Admassu B. (2021). The influence of weather conditions on body temperature, milk composition and yields of the free-ranging dromedary camels in Southeastern rangelands of Ethiopia. Cogent Food Agric..

[36] Saleh M.A., El-Mileegy I.M., El-Ela A.A. (2006). The interaction between temperature humidity index and some health parameters of sheep under the Egyptian oasis environment. Assiut Vet. Med. J..

[37] Van Saun R.J. (2023). Trace mineral metabolism: The maternal-fetal bond. Vet. Clin. Food Anim. Pract..

[38] Golombeski G.L. (2011). The Effect of Dietary Trace Minerals During Late Gestation on Health and Performance of the Dam and Progeny.

[39] Satheesan L., Dang A.K., Venkatesan R.T., Kamboj A. (2025). Temporal dynamics and climatic factors on milk yield, milk quality and mammary stress biomarkers in tropical Sahiwal cows (*Bos indicus*) reared under semi-intensive production system. Theor. Appl. Climatol..

[40] Faye B., Bengoumi M. (2018). Camel Clinical Biochemistry and Hematology.

[41] Emam R., Ghanem P.M.M., Abdel-Raof Y., El-Khaiat H.M., Elsayed A.A., Helal M.A. (2025). Gene Expression of Oxidative/Antioxidative Markers, VDR, CAMK4 and Ceruloplasmin in Baladi Sheep with Minerals Deficiency. Egypt. J. Vet. Sci..

[42] Bhalakiya N., Haque N., Patel P., Joshi P. (2019). Role of trace minerals in animal production and reproduction. Int. J. Livest. Res..

[43] Adeniji Y.A., Sanni M.O., Abdoun K.A., Samara E.M., Al-Badwi M.A., Bahadi M.A., Alhidary I.A., Al-Haidary A.A. (2020). Resilience of lambs to limited water availability without compromising their production performance. Animals.

[44] Prihandrijanti M., Azzizi V.T. (2023). Geospatial and temporal analysis of temperature-humidity index (THI) as climate mitigation tool in glamping site in Cimahi North, West Java, Indonesia. IOP Conference Series: Earth and Environmental Science.

[45] Țogoe D., Mincă N.A. (2024). The impact of heat stress on the physiological, productive, and reproductive status of dairy cows. Agriculture.

[46] Byrne L., Murphy R.A. (2022). Relative bioavailability of trace minerals in production animal nutrition: A review. Animals.

[47] Martínez-Morcillo S., Barrales I., Pérez-López M., Rodríguez F.S., Peinado J.S., Míguez-Santiyán M.P. (2024). Mineral and potentially toxic element profiles in the soil-feed-animal continuum: Implications for public, environmental, and livestock health in three pasture-based sheep farming systems. Sci. Total Environ..

[48] Cooke A.S., Machekano H., Gwiriri L.C., Tinsley J.H., Silva G.M., Nyamukondiwa C., Safalaoh A., Morgan E.R., Lee M.R. (2025). The nutritional feed gap: Seasonal variations in ruminant nutrition and knowledge gaps in relation to food security in Southern Africa. Food Secur..

[49] Damato A., Vianello F., Novelli E., Balzan S., Gianesella M., Giaretta E., Gabai G. (2022). Comprehensive review on the interactions of clay minerals with animal physiology and production. Front. Vet. Sci..

[50] Zou Z., Duley J.A., Cowley D.M., Reed S., Arachchige B.J., Shaw P.N., Bansal N. (2022). Comprehensive biochemical and proteomic characterization of seasonal Australian camel milk. Food Chem..

[51] Chainy G.B.N., Paital B., Dandapat J. (2016). An overview of seasonal changes in oxidative stress and antioxidant defence parameters in some invertebrate and vertebrate species. Scientifica.

[52] Tian W., Ma X., Liu H., Wang Z., Liu C., Xie C. (2025). Maternal Ferrous Sucrose Supplementation Improves Reproductive Performance of Sows and Hepatic Iron Stores of Neonatal Piglets. Animals.

[53] Harvey K.M., Cooke R.F., Marques R.d.S. (2021). Supplementing trace minerals to beef cows during gestation to enhance productive and health responses of the offspring. Animals.

